# Key factors associated with quality of postnatal care: a pooled analysis of 23 countries

**DOI:** 10.1016/j.eclinm.2023.102090

**Published:** 2023-07-20

**Authors:** Shuangyu Zhao, Yixuan Zhang, Angela Y. Xiao, Qiwei He, Kun Tang

**Affiliations:** a Vanke School of Public Health, Tsinghua University, No. 30, Shuangqing Road, Haidian District, Beijing, 100084, PR China; b School of Traffic and Transportation, Beijing Jiaotong University, No.3, Shangyuan Village, Haidian District, Beijing, 100044, PR China; c Institute of International Development Cooperation, Chinese Academy of International Trade and Economic Cooperation, Beijing, 100710, PR China

**Keywords:** Maternal and neonate health, Postnatal care, Quality of care, Key factors

## Abstract

**Background:**

Progress in reducing maternal and neonatal mortality, particularly in low-income and middle-income countries (LMICs) and regions, is insufficient to achieve the Sustainable Developmental Goals by 2030. High-quality postnatal care (PNC) for mothers and neonates is crucial for mothers and babies, yet it remains the most neglected intervention on the continuum of maternal and child care. We aimed to estimate the associations between observable factors and high-quality maternal and neonatal PNC in pooled and country-specific analyses.

**Methods:**

In this cross-sectional study, we used the most recent (2015–2022) Demographic and Health Surveys from 23 countries across Africa (n = 14), Southeast Asia (n = 3), Eastern Mediterranean (n = 2), Europe (n = 2), Americas (n = 1), and Western Pacific (n = 1). Women who, within the last 5 years, were aged 15–49 years at their last live birth that had delivered a singleton child were included. We identified eleven PNC behaviours recommended by the World Health Organization (WHO) to measure PNC quality, and applied thresholds to create binary outcomes for quality maternal and neonatal PNC. 15 factors were included in our analysis to assess their association with high-quality PNC for mothers and neonates in a series of single-adjusted and mutually adjusted logistic regression models, both in pooled and country-specific analysis. We also conducted two sets of subgroup analyses for place of residence and maternal age at last birth, and two sets of supplementary analyses to test the robustness of the results.

**Findings:**

Among 172,526 women and their most recent child, 41.42% (40.93–41.91) received quality maternal PNC while 42.34% (41.86–42.83) received quality neonatal PNC. In the pooled analysis, we found that the factors showing the strongest associations with quality maternal PNC were delivery by skilled birth attendants (SBAs) (OR: 4.92; 95% CI: 4.32–5.59), four or more antenatal care (ANC) visits (OR: 1.69, 1.58–1.81), and institutional delivery (OR: 1.61; 1.46–1.78). Consistent results were found for all factors of quality newborn care (e.g., delivery by SBA: OR, 4.25; 3.75–4.81; four or more ANC visits: OR, 1.83; 1.70–1.96) except institutional delivery. The association between these leading factors and PNC quality were broadly consistent across countries. Subgroup analyses and sensitivity analyses showed generally consistent results.

**Interpretation:**

Our study demonstrated that institutional delivery and frequent ANC visits had the strongest positive associations with quality PNC for both mothers and neonates. Our findings highlight that improvements to the quality of maternal and neonatal PNC in the LMICs we assessed are urgently needed to achieve ambitious maternal, newborn, and child health goals.

**Funding:**

China National Natural Science Foundation.

**Research in context**

**Evidence before this study**

Many studies have described the risk factors associated with the quality of health services in low-income and middle-income countries (LMICs) or regions. However, few have focused on the quality of postnatal care (PNC) for both mothers and neonates at a multi-country level while also following the latest guidelines for PNC proposed by the World Health Organization (WHO) to assess the quality of care. We searched Google Scholar, Web of Science, and PubMed for studies with the combination of the following terms: “postnatal care,” “maternal care,” “newborn care,” “postpartum,” “health seeking behaviour,” “quality of care,” and “risk factor,” “intervention,” “prevention,” “quality assessment,” with no date or language restrictions, with the last search carried out March 6, 2023. While negative associations between low maternal education, rural residence, high birth parity, non-health facility delivery, and maternal PNC coverage have been found in single country studies, past research failed to consider neonatal PNC, the quality of PNC, and cross-country heterogeneity. Although one study used timing, place, and assisted person to measure the quality of PNC in Uganda, this study only included women delivering in health facilities and did not follow the latest WHO guidelines.

**Added value of this study**

To our knowledge, this is the first multi-country study to systematically assess the relative significance of factors associated with the quality of maternal PNC and neonatal PNC. We used 11 behaviours to measure the quality of PNC, following the latest WHO guidelines. In both pooled and country-specific analyses, we identified the leading protective factors of high-quality PNC for mothers and neonates to be delivery by skilled birth attendants, institutional delivery, four or more antenatal care visits, and richest household wealth. Notably, we observed discordance in the association of institutional delivery with the quality of maternal PNC and neonatal PNC, in which institutional delivery was positively associated with high-quality maternal PNC but had no significant impact on quality neonatal PNC.

**Implications of all the available evidence**

Our findings suggest that prevention and intervention programmes targeting leading risk factors are needed to improve the quality of maternal and neonatal PNC in the LMICs we assessed. The available evidence highlights that quality improvement efforts should explicitly target low-income and other vulnerable populations to ensure that no one is left behind.

## Introduction

The postnatal period, defined as the first 42 days following childbirth, is a key phase for mothers and neonates.^1^ An estimated 529,000 women worldwide die each year from complications related to pregnancy and childbirth. Mortality can be extremely high in the postnatal period due to the significantly higher risk of mortality in the first week after the end of pregnancy, which may be up to 100 times higher than in the second year postpartum.^2^ In addition, poor quality care for mothers and neonates remains a major factor contributing to the approximate 2.9 million neonatal deaths per year, with the majority of these deaths taking place in the first 28 days of life.^3^

Postnatal care (PNC) services are a fundamental component of the continuum of maternal, neonatal and child care and pivotal in realising the Sustainable Development Goals (SDGs) on reproductive, maternal and child health, including the reduction of maternal mortality rates and the elimination of preventable neonatal deaths.^1^ However, despite the critical importance of this period for both maternal and neonatal survival, PNC remains the most neglected intervention on the continuum of maternal and child care.^4^ The most recent Countdown report showed that median coverage of postnatal care for mothers in low- and middle-income countries was 45%.^5^

To reduce mortality and enhance the health and survival of mothers and neonates during the postnatal period, efforts aimed at enhancing PNC must extend beyond coverage and survival alone to include quality of care. High-quality PNC is critical for the prevention and early detection of many potential causes of obstetric complications and neonatal deaths, such as hypothermia for neonates and maternal anaemia.^6^ Previous research has estimated that achieving 90% effective PNC coverage in sub-Saharan Africa could prevent 10–27% of all-cause neonatal deaths in the region.^3^ A recent systematic review identified the essential components of effective PNC, including breastfeeding, immediate examination of mothers and babies, skin-to skin care at birth for neonates, counselling for dangers of mothers and babies, immunisations for babies and other services.^6^

While associations between a specific risk factor and PNC coverage have been studied extensively,^7^ less is known about the factors influencing the quality of PNC. Furthermore, previous studies have quantified the effects of risk factors on maternal and neonatal PNC separately,^8^ or only focused on severe cases.^9^ However, the most recent WHO guidelines on PNC suggest that both maternal and neonatal care during the postnatal period are key strategies for ending preventable mortality.^1^ As opposed to mothers and babies being treated as separate entities, it has been proposed that the “mother–baby dyad” should be protected and supported simultaneously.^10^ Moreover, most studies have only focused on a single country, indicating that the cross-country heterogeneity of the relative significance of factors associated with positive PNC remains an underexplored topic.^11^

To fill the research gap on the continuum of maternal, newborn, and child care, understanding of the relative strengths of factors associated with PNC quality and their variation across countries is urgently needed. Using the most recent data from the Demographic and Health Survey (DHS), we selected a comprehensive set of factors associated with the quality of PNC for the mother–baby dyad and conducted a systematic analysis to assess their relative significance in 23 countries. In addition to pooled analyses, we present country-specific findings with the aim of informing the core intervention components needed to promote high-quality PNC services in each country.

## Methods

### Data sources and study population

**Fig. 1:**
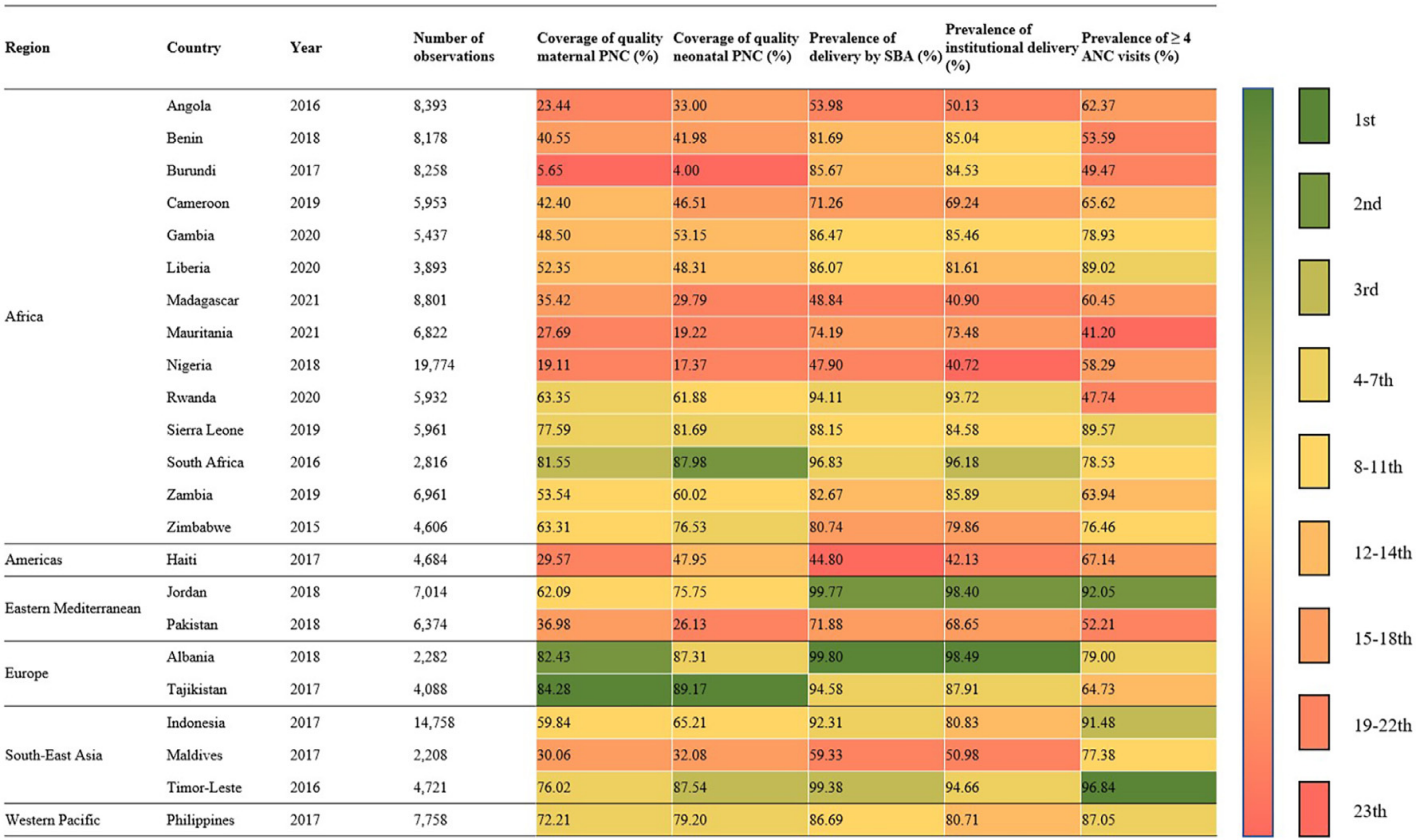
**Coverage of quality maternal PNC, neonatal PNC, and three essential services in 23 countries.** Note: Year stands for the survey year of the data included in the research; PNC indicates postnatal care; ANC indicates antenatal care; SBA indicates skilled birth attendant.

We extracted data from all DHS surveys conducted from 2015 to 2022 for each of the selected countries. As a result of several rigorous measurement examinations, the DHS, beginning with the DHS7 round implemented in approximately 2015, updated the questionnaire to include standardised indicators on whether neonates received immediate skin-to-skin contact, received PNC before facility discharge, and the four key components of a postnatal examination in the first two days after delivery. Because these indicators have only been collected in recent DHS7 surveys, data were only available from 23 of the countries across Africa (n = 14), South-East Asia (n = 3), Eastern Mediterranean (n = 2), Europe (n = 2), Americas (n = 1), and Western Pacific (n = 1) (Fig. 1). The 23 countries were Albania, Angola, Benin, Burundi, Cameroon, Gambia, Haiti, Indonesia, Jordan, Liberia, Madagascar, Maldives, Mauritania, Nigeria, Pakistan, Philippines, Rwanda, Sierra Leone, South Africa, Tajikistan, Timor-Leste, Zambia, and Zimbabwe. Analysis was based on the most recent DHS data available. Our study followed the Strengthening the Reporting of Observational Studies in Epidemiology (STROBE) reporting guidelines.

DHS surveys are nationally representative household surveys that collect detailed information of health seeking behaviours and the health conditions of women, children, and households. The sampling design involves a two-stage process, with a random selection of households from a list of enumeration areas (EAs) in the first stage, and a selection of all household members in the selected households in the second stage. We excluded surveys conducted before 2015 to ensure the data was up to date and to avoid missing or inconsistent measurements.^12^

**Fig. 2:**
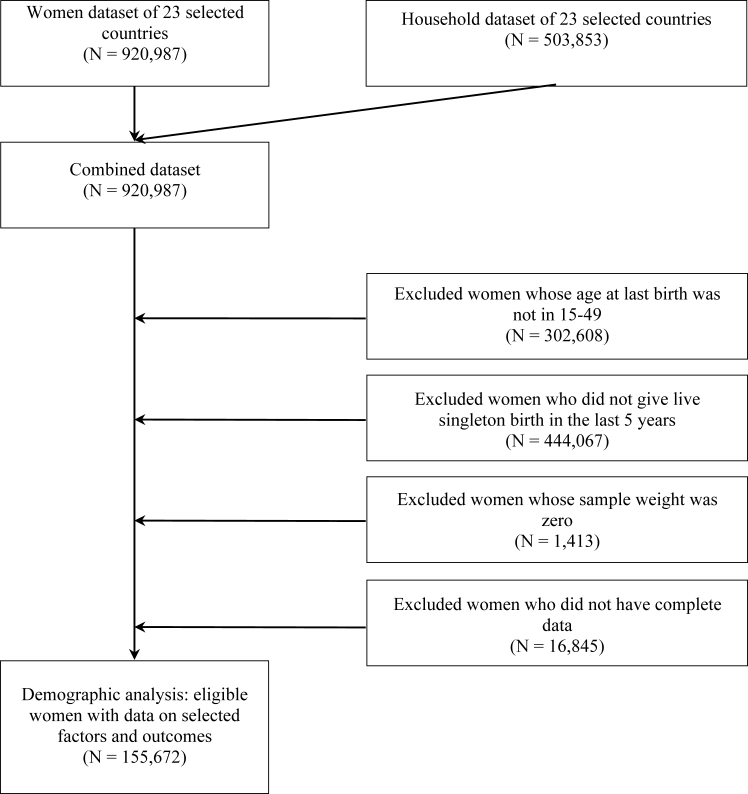
**Flow diagram showing exclusions and** **final sample sizes of the study population, using the most recent pooled Demographic Health Survey data since 2015.**

We sourced data from 920,937 women and their most recent child in 23 countries. Our study sample comprised of women (1) who had at least one live singleton birth in the past five years, (2) whose age was between 15 and 49 years at last childbirth, and (3) whose sampling weight was not equal to zero. We performed descriptive analysis on 172,526 women and used 155,672 women (excluding those with missing values) in our primary analysis (Fig. 2).

Written informed consent was obtained from all subjects conducted by DHS team. The data used in the study consisted of a publicly available de-identified dataset, which was retrieved from the DHS website with permission.

### Outcomes

Guided by the Lancet Global Health Commission framework on High-Quality Health Systems in the SDG Era^13^ and WHO Recommendations on Maternal and Newborn Care for a Positive Postnatal Experience,^1^ we assessed the availability of potential indicators of PNC quality in household surveys. High-quality PNC includes maternal care (e.g., maternal assessment and interventions for common physiological signs and symptoms), neonatal care (e.g., neonatal assessment, nutrition interventions, and infant growth and development), and health systems and health promotion interventions (e.g., scheduling for PNC visits). Our study focused exclusively on maternal and neonatal care to ensure the accuracy of quantifying measurements. The objective of this approach was to enable a meticulous and comprehensive analysis of the quality of PNC provided to mothers and their neonates among 23 countries.

Maintaining consistent measurement across all countries analysed, we found four indicators related to quality maternal PNC and nine indicators for quality neonatal PNC. The quality of maternal PNC was evaluated through a four-behaviour coding scheme, with the completion of at least two behaviours considered high-quality care in primary analysis. The four behaviours were: (1) receiving maternal PNC by a skilled birth assistant (SBA); (2) receiving maternal PNC in the first two days after the last delivery; (3) health provider counselling for the mother on breastfeeding in the first two days after the last delivery; and (4) health provider counselling for the mother on neonate dangers in first two days after the last delivery.

The quality of neonatal PNC was assessed using a nine-behaviour coding scheme: (1) receiving neonatal PNC by a SBA; (2) receiving neonatal PNC in the first two days after delivery; (3) placement of the neonate on the mother's bare skin after birth; (4) health provider examination of the neonate's cord in the first two days after delivery; (5) health provider examination of the neonate's temperature in the first two days after delivery; (6) health provider counselling for the mother on neonate dangers in the first two days after delivery; (7) health provider counselling for the mother on breastfeeding in the first two days after delivery; (8) health provider observation of the mother breastfeeding in the first two days after delivery; and (9) weighing of the neonate at birth. Completion of at least five of these nine behaviours was considered high-quality neonatal care in primary analysis.

Although these eleven indicators do not comprise the full range of essential PNC services and can only present a limited view of PNC quality, they have been recommended by WHO as essential components of PNC and are critical for maternal and newborn health.^14^

### Risk factors

Based on the existing literature, we identified a set of 15 observable risk factors and 3 covariates that are associated with the quality of PNC for mothers and neonates.^15^, ^16^, ^17^ A detailed list and description of all the risk factors can be found in Table 1.

**Table 1:** Definition of factors, covariates, and outcomes of maternal characteristics, neonatal characteristics, and household conditions associated with effective PNC for women and neonates.

Factors	Definition	Category	Reference group
Child's age	A classified variable describing the age of the woman's youngest child from 0 to 60 months old. Classified in the following 5 categories:	(1) 0–11	0–11
		(2) 12–23	
		(3) 24–35	
		(4) 36–47	
		(5) 48–59	
Child's sex	A binary variable describing the biological sex of the woman's last child in the 2 following categories:	(1) Male	Male
		(2) Female	
Maternal age at last birth	A classified variable describing the age at which the woman gave birth to her last child. Calculated based on the woman's documented birthday and the year of her last birth. Classified in the following 5 categories:	(1) 15–19	15–19
		(2) 20–34	
		(3) 35–49	
Maternal parity number	A classified variable describing the amount of woman's birth, and classified in the following 5 categories:	(1) 1	1
		(2) 2	
		(3) 3	
		(4) 4	
		(5) 5 and above	
Maternal educational attainment	A classified variable describing the woman's education level in the 2 following categories:	(1) Primary education and below	Primary education and below
		(2) Secondary education and above	
Frequency of newspaper or magazine reading	A binary variable describing if the woman has access to newspaper or magazine, and classified in the following 2 categories:	(1) Yes, if a woman reads newspapers or magazine at least once a week	No, otherwise
		(2) No, otherwise	
Frequency of radio listening	A binary variable describing if the woman has access to radio, and classified in the following 2 categories:	(1) Yes, if a woman listens to radio at least once a week	No, otherwise
		(2) No, otherwise	
Frequency of television watching	A binary variable describing if the woman has access to television, and classified in the following 2 categories:	(1) Yes, if a woman watches television at least once a week	No, otherwise
		(2) No, otherwise	
Owns a mobile telephone	A binary variable describing if the woman owns a mobile telephone, and classified in the following 2 categories:	(1) Yes	No
		(2) No	
Maternal age at first birth	A binary variable describing the woman's age at her first delivery:	(1) <18 years	<18 years
		(2) 18 years and above	
ANC visits	A classified variable describing the woman's ANC utilisation during her last pregnancy in the following 2 categories:	(1) 3 times and below	3 times and below
		(2) 4 times and above	
SBA at delivery	A binary variable describing if the woman's last delivery was assisted by a SBA, including physicians, nurses, and midwives:	(1) Yes	No
		(2) No	
Place of delivery	A binary variable describing the woman's place of last delivery in the following 2 categories:	(1) Institutional delivery, including public/private health facility, NGO sector facilities	Others
		(2) Others	
Marital status	A binary variable describing the woman's marital condition. Based on the survey launched by UNICEF, marital status classified in the 2 following categories:	(1) Married	Others, including unmarried, divorced, and separated
		(2) Others, including unmarried, divorced, and separated	
Head of household's sex	A binary variable describing the biological sex of the woman's head of household in the 2 following categories:	(1) Male	Male
		(2) Female	
Head of household's educational attainment	A classified variable describing the education level of the woman's head of household using the 3 following categories:	(1) Primary education and below	Primary education and below
		(2) Secondary education	
		(3) Higher education	
Household wealth index	A classified variable describing the level of household wealth, ascertained through a selected set of household assets (computer, television, telephone, and internet). Classified in the following 5 categories:	(1) Poorest	Poorest
		(2) Second	
		(3) Middle	
		(4) Forth	
		(5) Richest	
Place of residence	A binary variable describing the woman's place of residence in the following 2 categories:	(1) Urban	Rural
		(2) Rural	
Effective maternal PNC	A binary variable, was defined as Yes and coded as 1 if 2 or more of the following care were applied: (1) SBA provided maternal PNC, (2) maternal PNC was received in first 2 days after the last birth, (3) health provider counselled the mother on breastfeeding in first 2 days after the last birth, (4) health provider counselled the mother on neonate dangers in first 2 days. Otherwise, effective maternal PNC was defined as No and coded as 0.	(1) Yes	–
		(2) No	
Effective neonatal PNC	A binary variable, was defined as Yes and coded as 1 if 4 or more of the following care were applied: (1) SBA provided neonatal PNC, (2) neonatal PNC was received in first 2 days, (3) neonate was put on the mother's bare skin after birth, (4) health provider examined neonate's cord in first 2 days, (5) health provider examined neonate's temperature in first 2 days, (6) health provider counselled the mother on neonate dangers in first 2 days, (7) health provider counselled the mother on breastfeeding in first 2 days, (8) health provider observed the mother breastfeeding in first 2 days, (9) neonate was weighed at birth. Otherwise, effective neonatal PNC was defined as No and coded as 0.	(1) Yes	–
		(2) No	

The 15 risk factors were classified into three categories: household factors, maternal socioeconomic factors, and maternal care services. The four household factors were household wealth, place of residence, highest educational attainment of the head of household, and sex of the head of household. We identified a total of seven maternal socioeconomic factors: marital status, maternal age at first birth, maternal age at last birth, maternal education, frequency of newspaper/magazine reading, frequency of television watching, and ownership of a mobile phone. We identified three indicators of maternal care services related to the most recent birth: the number of antenatal care (ANC) visits, whether the delivery was institutional, and whether a SBA assisted the delivery. In addition, we collected three covariates that captured basic information about the most recent child: sex, age, and birth order.

### Statistical analysis

#### Regression models

We assessed the association of each factor with high-quality PNC for mothers and neonates by first pooling data from all countries and then separately for each country.^12^ To ensure our estimates were representative in both pooled and national level analyses, we included the sampling weight, clustering, and stratification variables provided by the DHS. Our sample was clustered at the primary sampling unit level to account for the interdependence of error terms within clusters and households.^12^ In pooled analyses, we reweighted observations to account for the country's population size as done previously,^18^ and used country fixed effects by adding a dichotomous variable for each country to account for the unobservable country-level factors.

For both pooled and country-specific analyses, we developed two sets of logistic regression models for each outcome. First, we performed a single-adjusted model for each risk factor, adjusting for the abovementioned three covariates. Second, we ran a fully adjusted model in which all risk factors and the three covariates were simultaneously included. Prior to multivariate analysis, we examined the multicollinearity using the variance inflation factor (VIF) (Appendix Table S1) and found that no variable had a VIF value above 10. Based on these models, we compared and ranked the risk factors according to their odds ratios (ORs). The factors with ORs significantly greater than one were identified as protective factors of quality maternal or neonatal PNC; those with ORs significantly less than one were identified as risk factors.

#### Subgroup and sensitivity analyses

We conducted subgroup analyses stratified by place of residence (rural or urban) and maternal age at last birth (aged 15–19, 20–34, or 35–49 years old). Previous studies have demonstrated that women living in rural areas are less likely to use PNC services compared to their urban counterparts, as in general distance to the nearest health facility is greater in rural areas.^19^ Different maternal ages at pregnancy may be linked with PNC utilisation in varying ways. Adolescent mothers have been found to demonstrate lower motivation in utilising the continuum of health services, including postnatal services,^20^ and may be more likely to have lower socioeconomic status.^21^ Therefore, we stratified the sample by maternal age at last birth to investigate the relative importance of risk factors in each subgroup.

We conducted two sets of sensitivity analyses for each outcome variable: first, we refined our definition of high-quality PNC by adjusting the cut-off thresholds for PNC behaviours. Our aim with this approach was to better discern the level of PNC quality that mothers received. In primary analyses, high-quality maternal PNC was measured as standard for women who demonstrated two or more PNC behaviours. In supplementary analyses, three behaviours were required to identify quality maternal PNC. Similarly, for assessing quality neonatal PNC, primary analyses required meeting at least five out of nine behaviours, while the supplementary analyses required meeting six out of nine behaviours (Appendix Tables S2 and S3).

Second, a stricter VIF criterion was used to avoid potential multicollinearity. The correlation matrix revealed a high correlation between institutional delivery and delivery assisted by SBAs. Therefore, we re-estimated the fully adjusted models by separately removing delivery by SBAs and institutional delivery.

All analyses were performed using Stata version 17 (StataCorp). Following previous practice, we assumed that the data were missing at random.^22^ As the missing rate for all variables was less than 10%, we excluded observations with missing data. All statistical tests were 2-tailed, and P < .05 was considered statistically significant.

### Role of the funding source

The funder did not have any role in the study design, data analysis, data interpretation, report writing, or publication submission.

## Results

### Postnatal services utilisation

A total of 172,526 women from 23 countries who were aged 15–49 years at last childbirth with at least one live singleton birth in the past five years were included in the descriptive analysis, and 155,672 were included in the regression analysis after excluding missing values (Fig. 2). Basic characteristics of respondents are displayed in Table 2, and country-specific details are shown in Fig. 1. Across the 23 countries with available data, 41.42% of respondents received quality maternal PNC, and 42.34% of respondents’ last children received quality neonatal PNC. Tajikistan had the highest coverage of both maternal PNC (84.28%) and neonatal PNC (89.17%), while Burundi ranked last, with only 5.65% of mothers receiving maternal PNC and 4.00% of neonates receiving neonatal PNC.

**Table 2:** Distribution of effective PNC by selected factors among women aged 15–49 years, using the most recent Demographic Health Surveys pooled across 23 countries.

Factor	Prevalence%, (95% CI)		
	Women observed, No. (%)	Effective maternal PNC	Effective neonatal PNC
Total sample for pooled analysis across 24 countries	172,526 (100)	41.42 (40.93–41.91)	42.34 (41.86–42.83)
Child's age, months			
0–11	49,414 (28.65)	24.81 (24.15–25.48)	23.98 (23.34–24.63)
23-Dec	45,066 (26.12)	24.97 (24.29–25.67)	24.56 (23.90–25.24)
24–35	34,384 (19.93)	19.44 (18.83–20.07)	19.79 (19.18–20.41)
36–47	24,546 (14.23)	16.58 (16.00–17.18)	16.96 (16.38–17.55)
48–59	19,106 (11.07)	14.20 (13.65–14.77)	14.71 (14.16–15.28)
Child's sex			
Male	87,859 (50.93)	51.48 (50.69–52.26)	51.47 (50.69–52.24)
Female	84,657 (49.07)	48.52 (47.74–49.31)	48.53 (47.76–49.31)
Place of residence			
Urban	65,642 (38.05)	53.57 (52.78–54.36)	53.13 (52.36–53.90)
Rural	106,874 (61.95)	46.43 (45.64–47.22)	46.87 (46.10–47.64)
Maternal age at first birth, y			
<18	39,932 (23.15)	12.79 (12.32–13.27)	12.89 (12.43–13.37)
≥18	118,727 (68.82)	87.21 (86.73–87.68)	87.11 (86.63–87.57)
Missing	13,857 (8.03)	0.00 (0.00–0.00)	0.00 (0.00–0.00)
Maternal age at last birth, y			
15–19	18,635 (10.80)	7.01 (6.64–7.39)	6.94 (6.58–7.31)
20–34	121,309 (70.32)	74.11 (73.43–74.79)	73.44 (72.76–74.11)
35–49	32,572 (18.88)	18.88 (18.27–19.50)	19.62 (19.02–20.25)
Maternal education attainment			
Primary and below	95,602 (55.42)	25.78 (25.11–26.46)	25.49 (24.84–26.16)
Secondary and higher	76,914 (44.58)	74.22 (73.54–74.89)	74.51 (73.84–75.16)
Frequency of newspaper/magazine reading			
Seldom	146,189 (84.74)	89.8 (88.70–89.62)	88.97 (88.51–89.42)
Once a week at least	12,432 (7.21)	10.79 (10.34–11.26)	11.00 (10.56–11.46)
Missing	13,895 (8.05)	0.03 (0.02–0.08)	0.03 (0.01–0.07)
Frequency of radio listening			
Seldom	115,332 (66.85)	76.12 (75.48–76.73)	75.97 (75.35–76.58)
Once a week at least	43,321 (25.11)	23.88 (23.26–24.52)	24.00 (23.39–24.63)
Missing	13,863 (8.04)	0.00 (0.00–0.03)	0.03 (0.01–0.10)
Frequency of television watching			
No	95,699 (55.47)	27.37 (26.71–28.03)	26.27 (25.64–26.91)
Yes	62,945 (36.49)	72.59 (71.93–73.25)	73.68 (73.04–74.31)
Missing	13,872 (8.04)	0.04 (0.02–0.08)	0.05 (0.02–0.10)
Owns a mobile phone			
No	67,965 (39.40)	22.49 (21.86–23.13)	21.75 (21.16–22.38)
Yes	90,678 (52.56)	77.47 (76.83–78.10)	78.19 (77.57–78.80)
Missing	13,873 (8.04)	0.04 (0.02–0.10)	0.04 (0.02–0.10)
Skilled birth attendant at delivery			
No	39,772 (23.05)	4.29 (4.01–4.60)	6.02 (5.69–6.37)
Yes	118,877 (68.91)	95.70 (95.39–95.99)	93.97 (93.62–94.31)
Missing	13,867 (8.04)	0.01 (0.00–0.06)	0.01 (0.00–0.06)
ANC visits			
<4	50,674 (29.38)	11.46 (11.02–11.92)	10.89 (10.48–11.32)
≥4	107,173 (62.12)	87.17 (86.68–87.64)	87.73 (87.27–88.18)
Missing	14,669 (8.50)	1.37 (1.19–1.58)	1.38 (1.20–1.58)
Institutional delivery			
No	47,139 (27.32)	11.62 (11.14–12.12)	14.75 (14.22–15.29)
Yes	113,651 (65.88)	88.38 (87.88–88.86)	85.25 (84.71–85.78)
Missing	11,726 (6.80)	0.00 (0.00–0.00)	0.00 (0.00–0.00)
Marital status			
Married	39,164 (22.70)	14.86 (14.40–15.34)	15.40 (14.94–15.88)
Others	119,495 (69.27)	85.14 (84.66–85.60)	84.60 (84.12–85.06)
Missing	13,857 (8.03)	0.00 (0.00–0.00)	0.00 (0.00–0.00)
Maternal parity number			
1	40,326 (23.37)	31.56 (30.81–32.30)	31.03 (30.30–31.76)
2	37,963 (22.01)	29.87 (29.14–30.61)	30.23 (29.51–30.96)
3	29,865 (17.31)	18.46 (17.87–19.08)	18.74 (18.15–19.35)
4	21,402 (12.41)	9.52 (9.09–9.97)	9.43 (9.02–9.86)
5 and above	42,960 (24.90)	10.59 (10.17–11.04)	10.57 (10.16–10.99)
Head of household's sex			
Male	136,834 (79.32)	86.31 (85.81–86.80)	86.34 (85.85–86.82)
Female	35,682 (20.68)	13.69 (13.20–14.19)	13.66 (13.18–14.15)
Head of household's education attainment			
Primary and below	89,948 (52.14)	38.75 (37.98–39.52)	38.46 (37.71–39.22)
Secondary and higher	68,697 (39.82)	61.23 (60.46–62.00)	61.50 (60.75–62.25)
Missing	13,871 (8.04)	0.02 (0.01–0.05)	0.04 (0.02–0.08)
Household wealth index			
Poorest	42,209 (24.46)	14.00 (13.50–14.51)	14.80 (14.30–15.30)
Second	37,822 (21.92)	16.82 (16.26–17.40)	17.30 (16.74–17.88)
Middle	35,291 (20.46)	19.89 (19.28–20.51)	19.90 (19.30–20.52)
Fourth	30,701 (17.80)	23.43 (22.75–24.13)	22.93 (22.27–23.61)
Richest	26,493 (15.36)	25.86 (25.15–26.58)	25.07 (24.38–25.77)

**Fig. 3:**
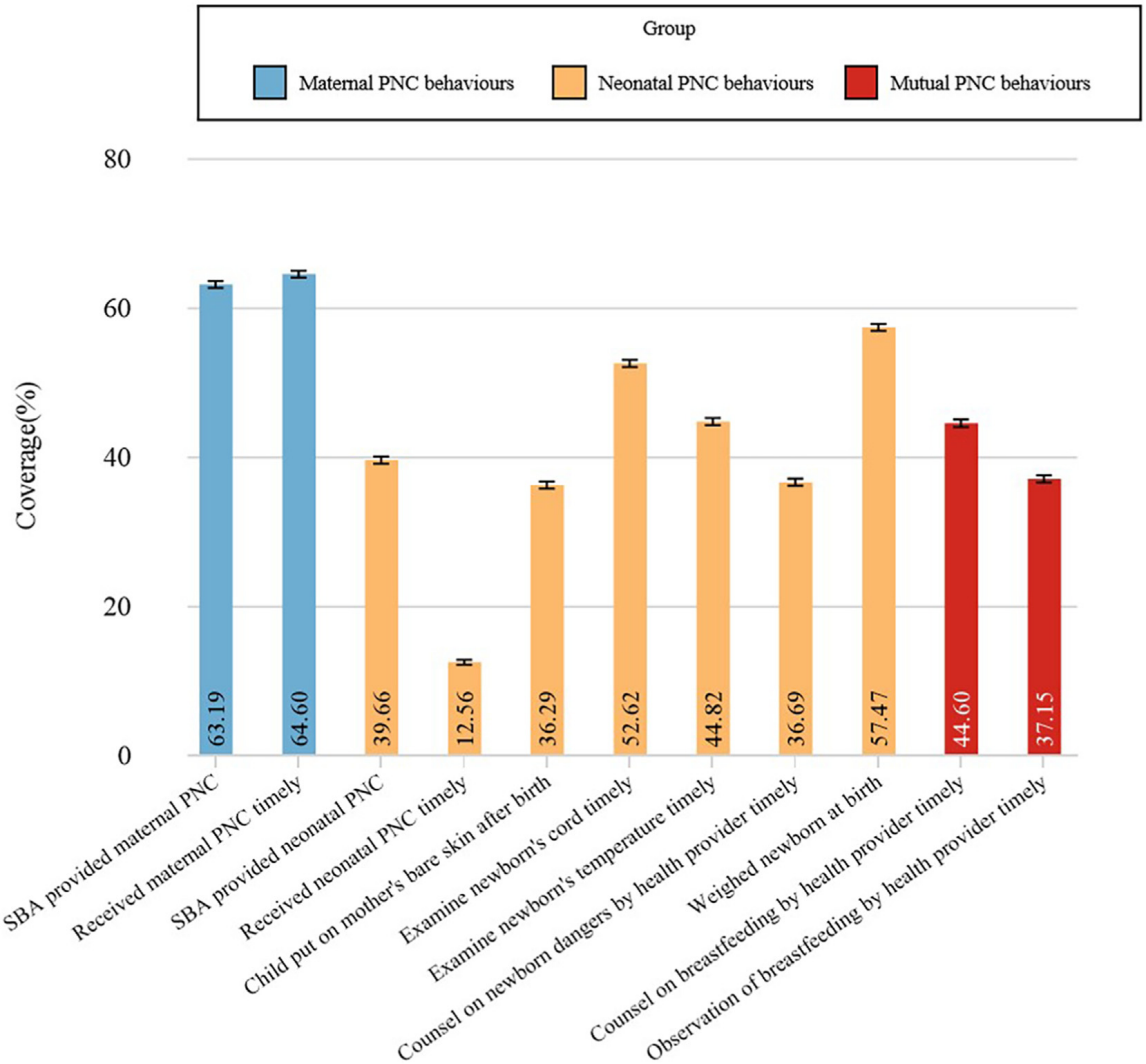
**Coverage of 11 selected PNC behaviours among women aged 15–49 years, using the most recent Demographic Health Surveys pooled across 23 countries (%).** Note: Received maternal PNC timely indicates that the mother received maternal PNC in the first two days after delivery; Received neonatal PNC timely indicates that the neonate received neonatal PNC in the first two days after birth; Examine neonate's cord timely indicates that examination of the neonate's cord occurred in the first two days after birth; Examine neonate's temperature timely indicates that examination of the neonate's temperature occurred in the first two days after birth; Counsel on neonate dangers by health provider timely indicates that the mother received health provider counselling on neonate dangers in first two days after the last birth; Counsel on breastfeeding by health provider timely indicates that the mother received health provider counselling on breastfeeding in first two days after the last birth; Observe breastfeeding by health provider timely indicates that a health provider observed the mother breastfeeding in first two days after the last birth; PNC indicates postnatal care; SBA indicates skilled birth attendant.

Eleven PNC services were studied in the research. Between the two exclusive behaviours for mothers, receiving maternal PNC within the first two days was the most prevalent (64.60%) (Fig. 3). Among the seven exclusive behaviours for neonatal PNC, the most prevalent was weighing of the neonate at birth (57.47%). Most other behaviours displayed high coverage, ranging from 36.69% for receiving health provider counselling on neonate dangers in the first two days after delivery to 52.62% for examination of the neonate's cord in the first two days after birth. Receiving neonatal PNC in the first two days after delivery had the lowest coverage (12.56%). Coverage of the mutual measures was around the median high. Our study found that 55.76% of mothers received quality maternal PNC, and 52.84% of babies received quality neonatal PNC (Appendix Tables S2 and S3).

### Pooled analyses

**Fig. 4:**
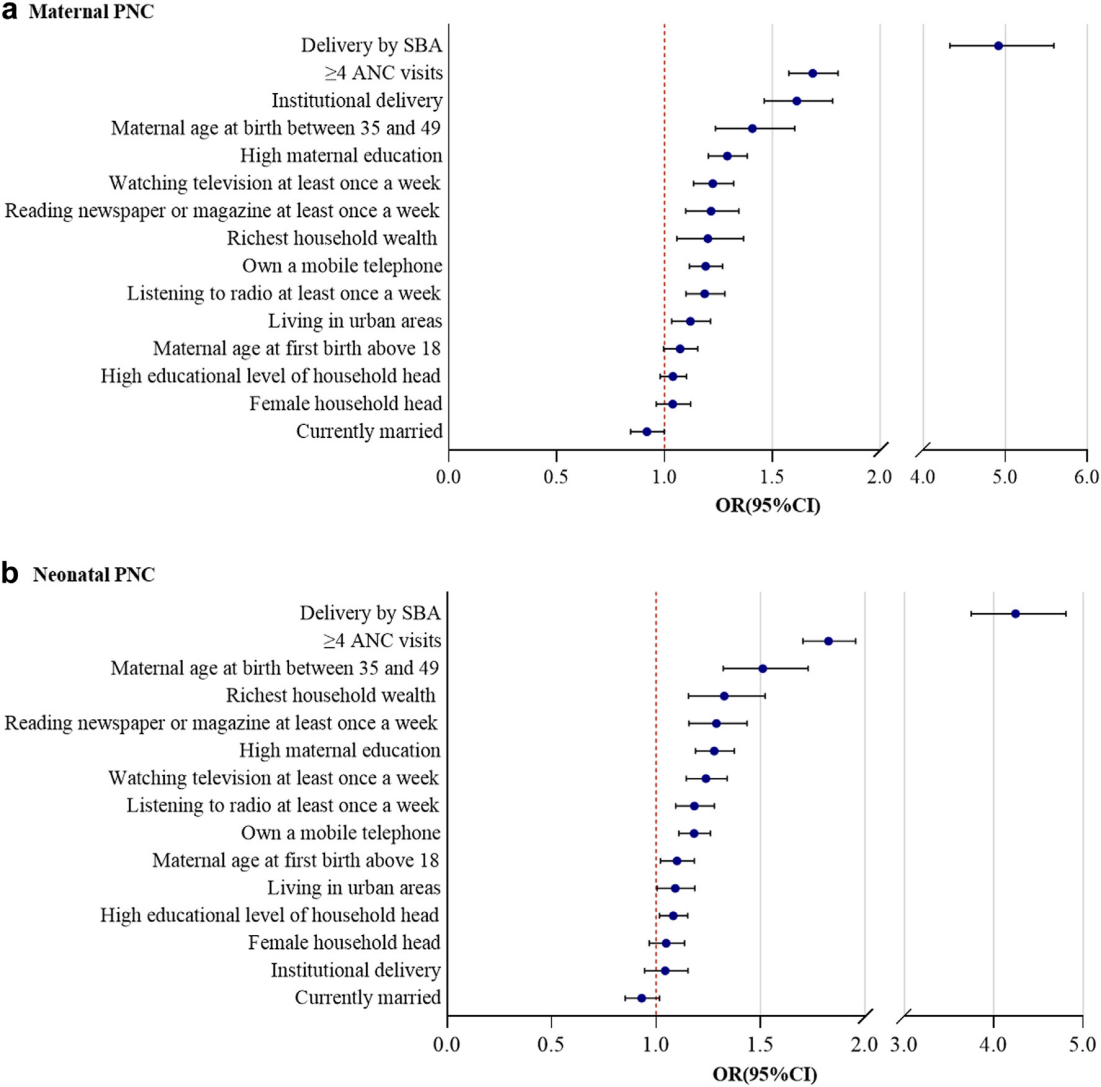
**Relative ranking of 15 factors associated with quality PNC from fully adjusted models on maternal care and neonatal care.** Note: High maternal education indicates the woman received secondary education or above; High education level of household head indicates the head of the woman's household received secondary education or above; PNC indicates postnatal care; ANC indicates antenatal care; SBA indicates skilled birth attendant; OR indicates odds ratio.

**Appendix Fig. S1:**
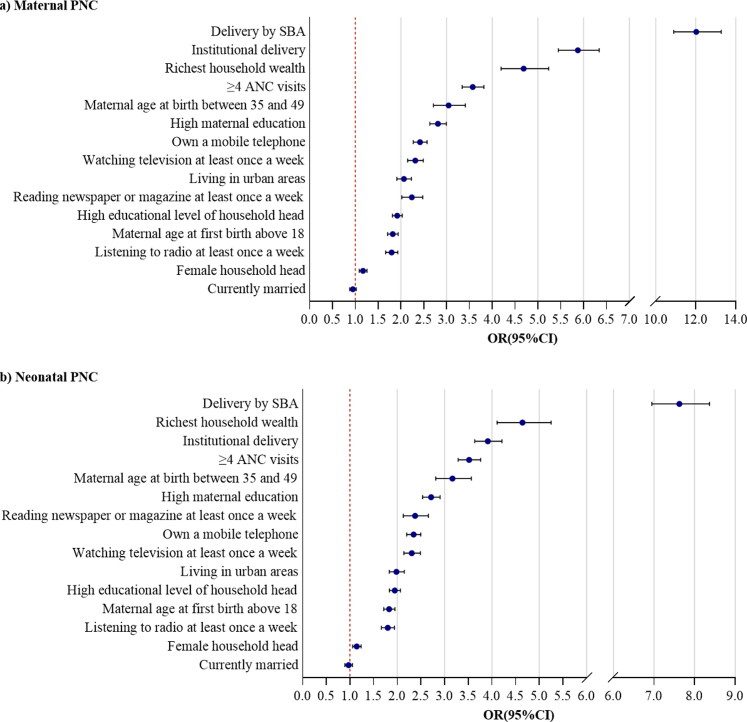
**Relative ranking of 15 factors associated with quality PNC from supplementary analysis of single-adjusted models on maternal care and neonatal care.** Note: High maternal education indicates the woman received secondary education or above; High education level of household head indicates the head of the woman’s household received secondary education or above; PNC indicates postnatal care; ANC indicates antenatal care; SBA indicates skilled birth attendant; OR indicates odds ratio.

We present the relative rankings of the pooled analyses in Appendix Fig. S1 (single-adjusted model) and Fig. 4 (mutually adjusted model). In single-adjusted models for high-quality maternal PNC, all factors except for currently married were significantly associated with higher odds of receiving quality PNC (Appendix Fig. S1A). Delivery by SBA had the strongest association with quality PNC (OR: 12.03, 95% CI: 10.90–13.28), followed by institutional delivery (OR: 5.87, 95% CI: 5.44–6.34) and richest household wealth (OR: 4.69, 95% CI: 4.19–5.24). Similar results were observed in quality neonatal PNC (Appendix Fig. S1B), where delivery by SBA, richest household wealth, and institutional delivery were the top three influencing factors. However, the magnitude of ORs for neonatal PNC was smaller than those for maternal PNC (e.g., institutional delivery, OR: 3.91, 95% CI: 3.64–4.21 for neonatal PNC, compared to OR: 5.87, 95% CI: 5.44–6.34 for maternal PNC).

The magnitude of associations substantially attenuated for most factors in the fully adjusted model. However, 11 factors related to high-quality maternal PNC remained highly statistically significant (Fig. 4A). When considering all other factors, delivery by SBA still ranked first (OR, 4.92, 95% CI: 4.32–5.59), followed by four or more ANC visits (OR: 1.69, 95% CI: 1.58–1.81), institutional delivery (OR: 1.61; 95% CI: 1.46–1.78), and maternal age at last birth between 35 and 49 (OR: 1.41; 95% CI: 1.20–1.64). For the quality of neonatal PNC, we found 12 factors significantly associated with higher odds of high-quality neonatal PNC (Fig. 4B), including delivery by SBA (OR: 4.25, 95% CI: 3.75–4.81), four or more ANC visits (OR: 1.83, 95% CI: 1.70–1.96), maternal age at last birth between 35 and 49 (OR: 1.51, 95% CI: 1.32–1.73), and richest household wealth (OR: 1.33, 95% CI: 1.16–1.52). Notably, institutional delivery ranked second to last and had no statistical significance (OR: 1.04, 95% CI: 0.95–1.15).

### Country-specific analyses

**Fig. 5:**
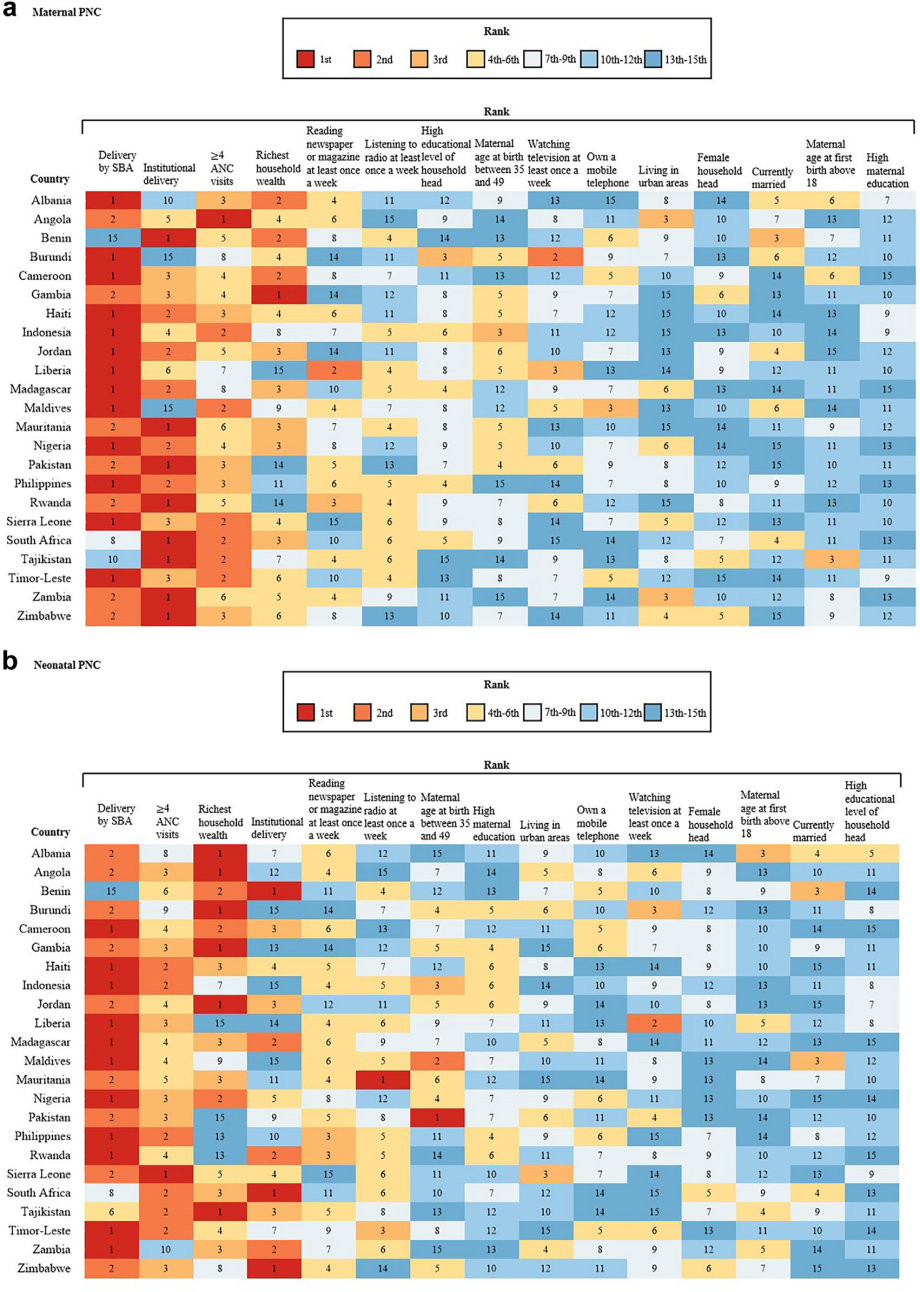
**Country-specific ranking of 15 factors associated with quality PNC from fully adjusted models on maternal care and neonatal care in 23 countries.** Note: High maternal education indicates the woman received secondary education or above; High education level of household head indicates the head of the woman's household received secondary education or above; PNC indicates postnatal care; ANC indicates antenatal care; SBA indicates skilled birth attendant; OR indicates odds ratio.

**Appendix Fig. S2:**
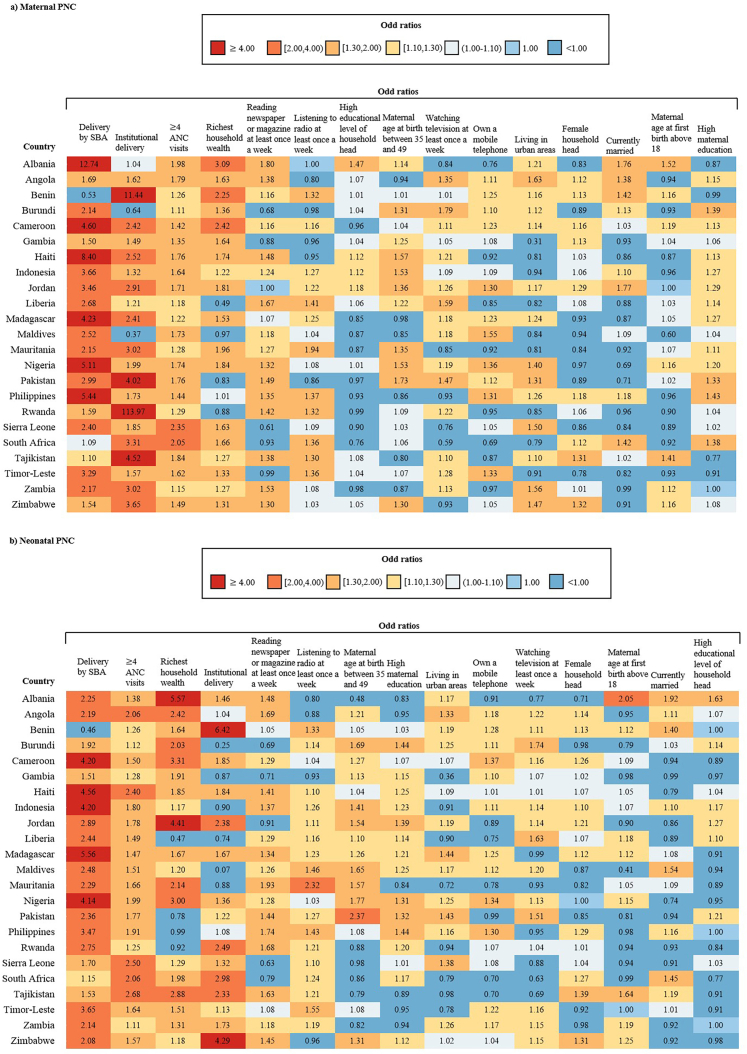
**Country-specific odds ratios for 15 factors associated with quality PNC from fully adjusted models on maternal care and neonatal care in 23 countries.** Note: High maternal education indicates the woman received secondary education or above; High education level of household head indicates the head of the woman’s household received secondary education or above; PNC indicates postnatal care; ANC indicates antenatal care; SBA indicates skilled birth attendant; OR indicates odds ratio.

We used multivariable logistic regression models to estimate the association between 15 factors and high-quality PNC for mothers and neonates in each country. Delivery by SBA had the strongest association with both quality maternal and neonatal PNC, ranking first or second in most countries (Fig. 5). However, it ranked last in Benin, tenth in Tajikistan, and eighth in South Africa for quality maternal PNC. Institutional delivery, four or more ANC visits, and richest household wealth were also strongly associated with quality maternal and neonatal PNC in most countries, generally ranking within the top four protective risk factors. Other factors, such as high exposure to mass media, advanced maternal age (between 35 and 49 years) at last birth, and urban residence, showed large variations in rankings across countries. The magnitudes of ORs for most factors were also heterogeneous (Appendix Fig. S2). For example, for high-quality maternal PNC, the magnitudes of ORs for delivery by SBA ranged from 0.53 in Benin to 12.74 in Albania, while the magnitudes of ORs for institutional delivery ranged from 0.37 in Maldives to 113.97 in Rwanda. In contrast, for quality neonatal PNC, the magnitude of ORs for four or more ANC visits was almost consistently within the range of 1.00–2.50.

Many countries with high levels of PNC coverage for mothers and neonates had high levels of healthcare utilisation, including delivery by SBA, institutional delivery, and four or more ANC visits (Fig. 1). These three factors were identified as the most crucial protective factors in our study. For example, more than 80% of women in South Africa, Sierra Leone, Timor-Leste, Tajikistan, Albania, and the Philippines received quality maternal PNC, which coincided with high utilisation rates of the three aforementioned services. Some countries had both low quality PNC and low utilisation of the three aforementioned services (Fig. 1). In Madagascar, Angola, Nigeria, and Haiti, less than 40% of respondents and their last child received high-quality PNC, and most reported low care-seeking behaviours. We also found that even if only one of the three health services had low coverage, the coverage of high-quality PNC for both mothers and neonates could be low (Fig. 1). For example, only 5.65% of women received quality PNC in Burundi, and although both delivery by SBA and institutional delivery were high (85.67% and 84.53%, respectively), only 49.47% attended four or more ANC visits.

### Subgroup and sensitivity analyses

**Appendix Fig. S3:**
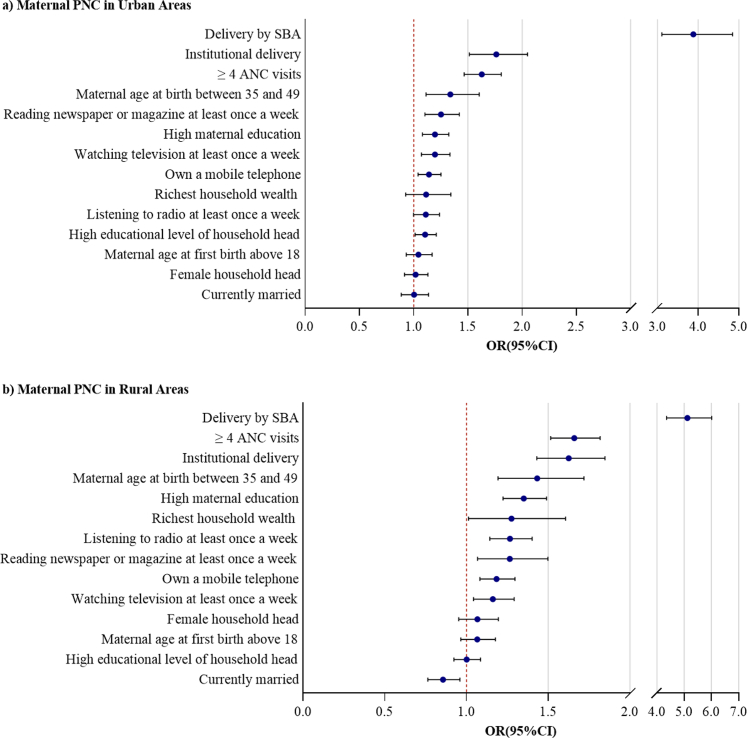
**Relative ranking of factors associated with maternal PNC from supplementary analysis of fully adjusted models, stratified by place of residence.** Note: High maternal education indicates the woman received secondary education or above; High education level of household head indicates the head of the woman’s household received secondary education or above; PNC indicates postnatal care; ANC indicates antenatal care; SBA indicates skilled birth attendant; OR indicates odds ratio.

**Appendix Fig. S4:**
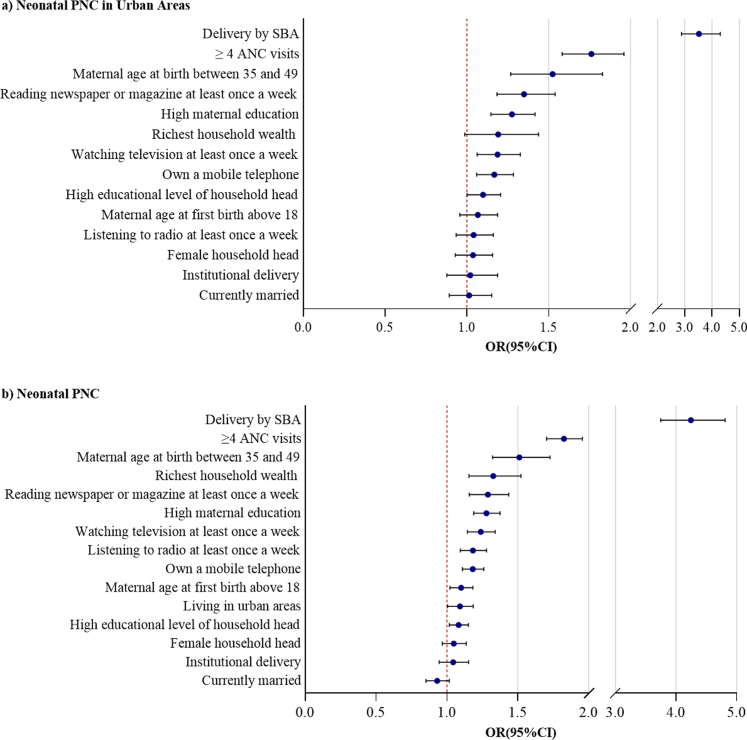
**Relative ranking of factors associated with neonatal PNC from supplementary analysis of fully adjusted models, stratified by place of residence.** Note: High maternal education indicates the woman received secondary education or above; High education level of household head indicates the head of the woman’s household received secondary education or above; PNC indicates postnatal care; ANC indicates antenatal care; SBA indicates skilled birth attendant; OR indicates odds ratio.

**Appendix Fig. S5:**
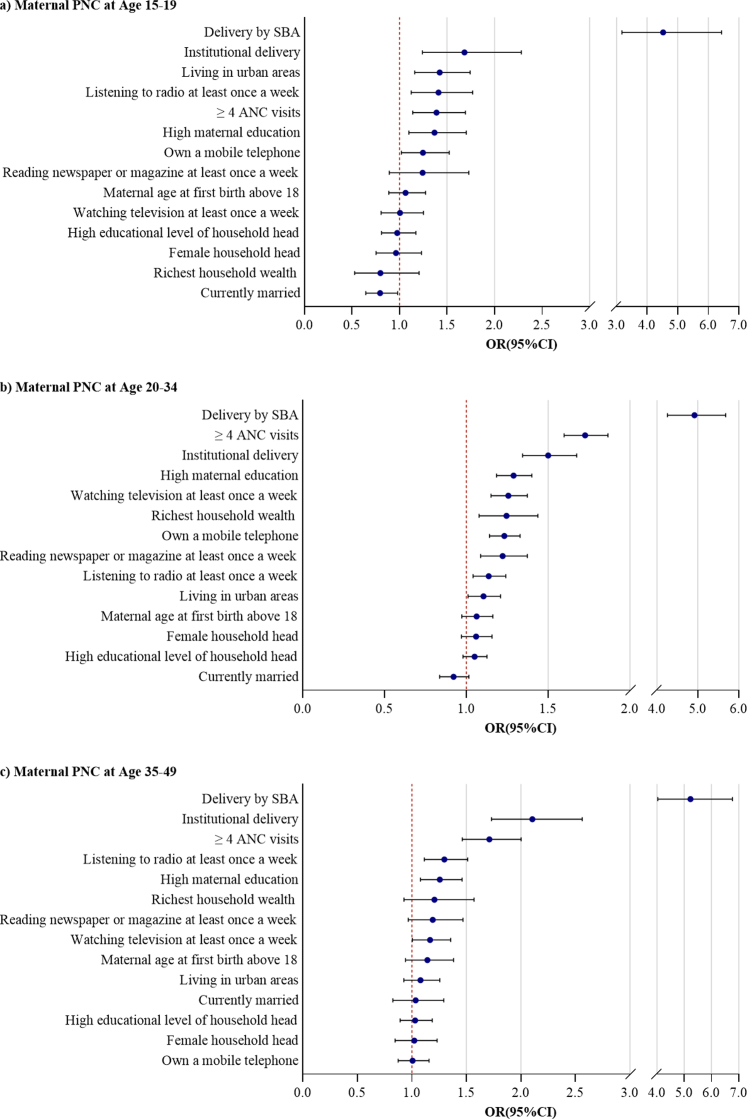
**Relative ranking of factors associated with maternal PNC from supplementary analysis of fully adjusted models, stratified by maternal age at last birth.** Note: High maternal education indicates the woman received secondary education or above; High education level of household head indicates the head of the woman’s household received secondary education or above; PNC indicates postnatal care; ANC indicates antenatal care; SBA indicates skilled birth attendant; OR indicates odds ratio.

**Appendix Fig. S6:**
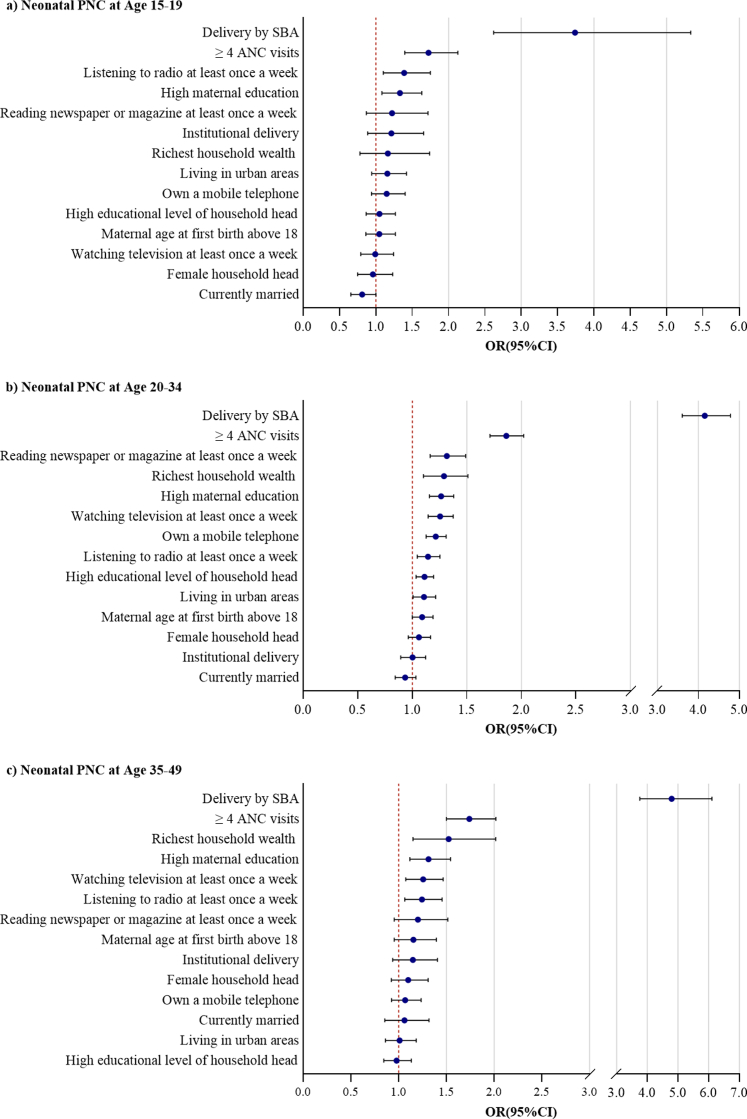
**Relative ranking of factors associated with neonatal PNC from supplementary analysis of fully adjusted models, stratified by maternal age at last birth.** Note: High maternal education indicates the woman received secondary education or above; High education level of household head indicates the head of the woman’s household received secondary education or above; PNC indicates postnatal care; ANC indicates antenatal care; SBA indicates skilled birth attendant; OR indicates odds ratio.

We conducted four sets of supplementary analyses. Firstly, we developed subgroup analyses stratified by place of residence. The results were similar to primary analyses, but differences between the urban and rural residence groups were found (Appendix Figs. S3 and S4). When stratifying the regression models by maternal age at last birth, risk factors remained largely unchanged, with delivery by SBA remaining the top leading risk factor (Appendix Figs. S5 and S6).

**Appendix Fig. S7:**
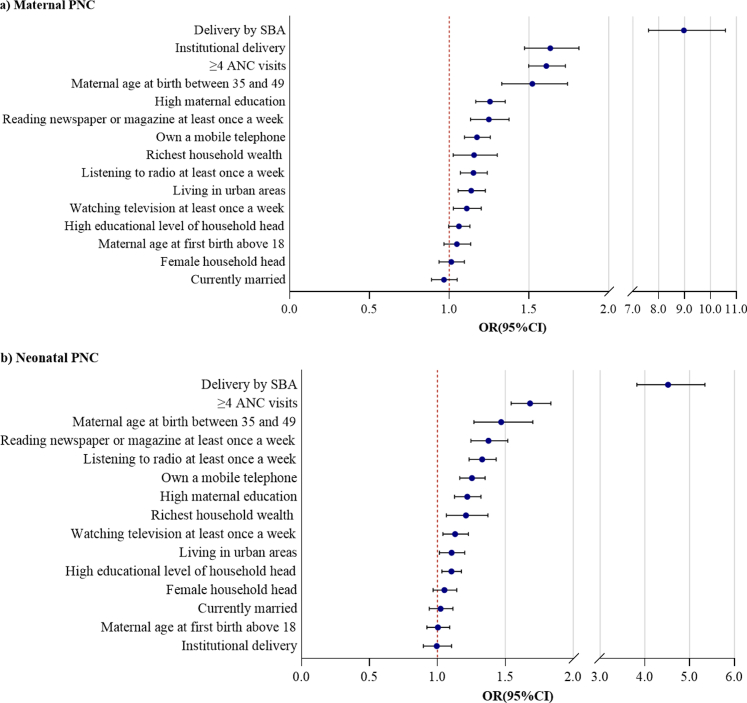
**Relative ranking of 15 factors associated with quality PNC from supplementary analysis of fully adjusted models on maternal care and neonatal care, re-scored by number of PNC behaviours.** Note: 1. Maternal PNC behaviours adopted in the construction of PNC include received maternal PNC by a SBA, received maternal PNC in first two days after the last birth, health provider counselling on neonate dangers in first two days after the last birth, and health provider counselling on breastfeeding in first two days after the last birth; 2. Neonatal PNC behaviours adopted in the construction of PNC quality include SBA at neonatal postnatal care, received neonatal PNC in first two days after the last birth, placement of the neonate put on the mother's bare skin after birth, examination of the neonate's cord by a health provider in first two days after the last birth, examination of the neonate's temperature by a health provider in first two days after the last birth, health provider counselling for the mother on neonate dangers in first two days after the last birth, health provider counselling for the mother on breastfeeding in the first two days after delivery, health provider observation of the mother breastfeeding in the first two days after delivery, and weighing of the neonate at birth; 3. Postnatal care quality was calculated using each cut-off value which requires three fourths of total maternal PNC and six ninths of total neonate PNC, accounting for the estimated coefficients from the mutually adjusted model; High maternal education indicates the woman received secondary education or above; High education level of household head indicates the head of the woman’s household received secondary education or above; PNC indicates postnatal care; ANC indicates antenatal care; SBA indicates skilled birth attendant; OR indicates odds ratio.

After refining the definition of quality PNC, we found that as the cut-off threshold lifted, the prevalence of high-quality PNC decreased (Appendix Tables S2 and S3). The rankings remained consistent in the fully adjusted model with the new cut-off threshold (Appendix Fig. S7).

**Appendix Fig. S8:**
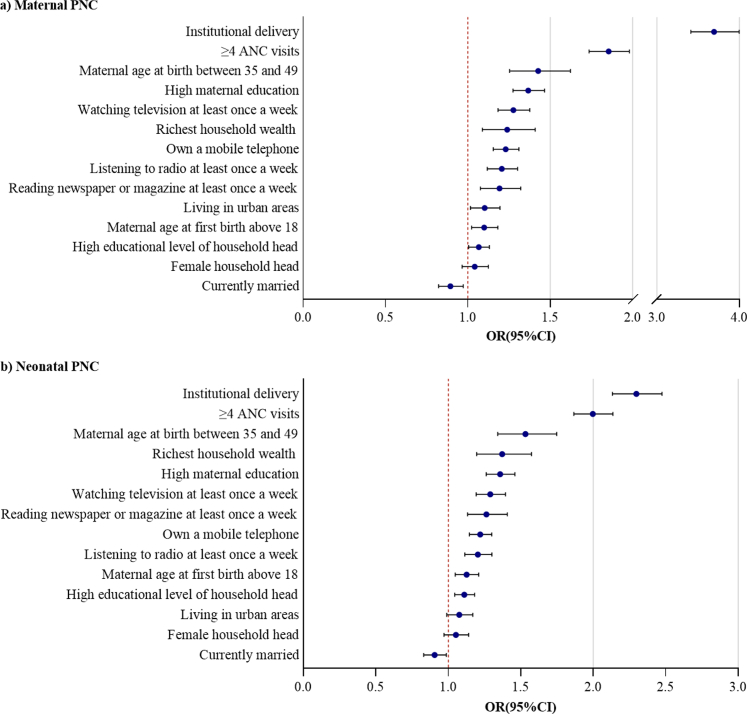
**Relative ranking of 14 factors associated with quality PNC from supplementary analysis of fully adjusted models on maternal care and neonatal care, excluding delivery by SBA.** Note: High maternal education indicates the woman received secondary education or above; High education level of household head indicates the head of the woman’s household received secondary education or above; PNC indicates postnatal care; ANC indicates antenatal care; SBA indicates skilled birth attendant; OR indicates odds ratio.

**Appendix Fig. S9:**
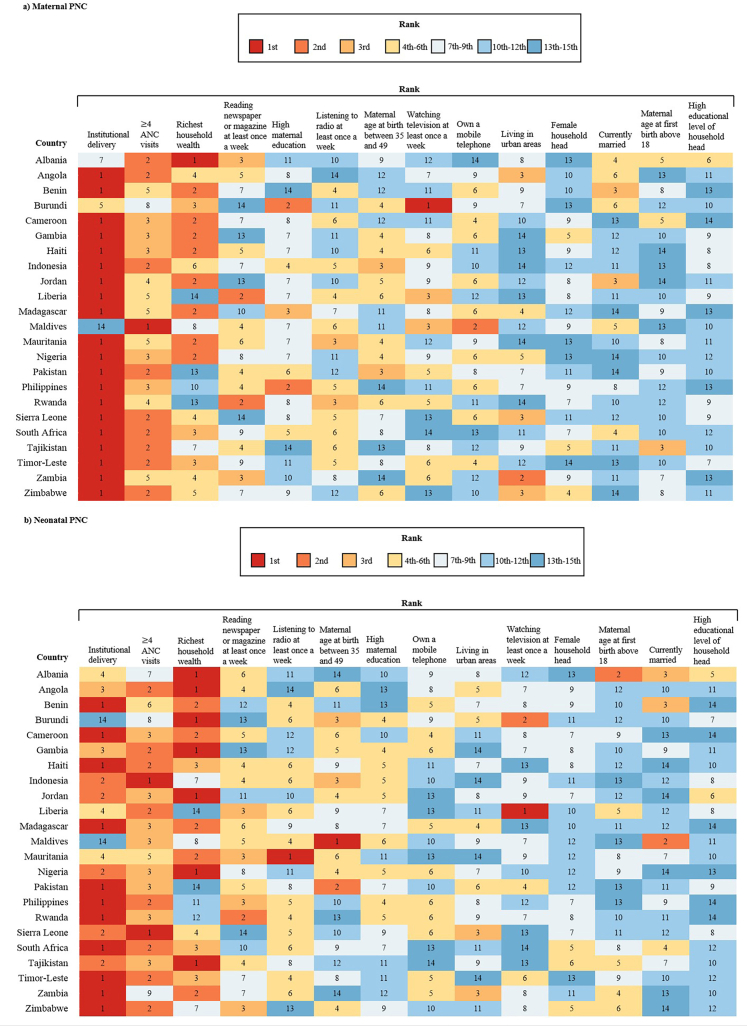
**Country-specific ranking of 14 factors associated with quality PNC from supplementary analysis of fully adjusted models on maternal care and neonatal care in 23 Countries, excluding delivery by SBA.** Note: High maternal education indicates the woman received secondary education or above; High education level of household head indicates the head of the woman’s household received secondary education or above; PNC indicates postnatal care; ANC indicates antenatal care; SBA indicates skilled birth attendant; OR indicates odds ratio.

**Appendix Fig. S10:**
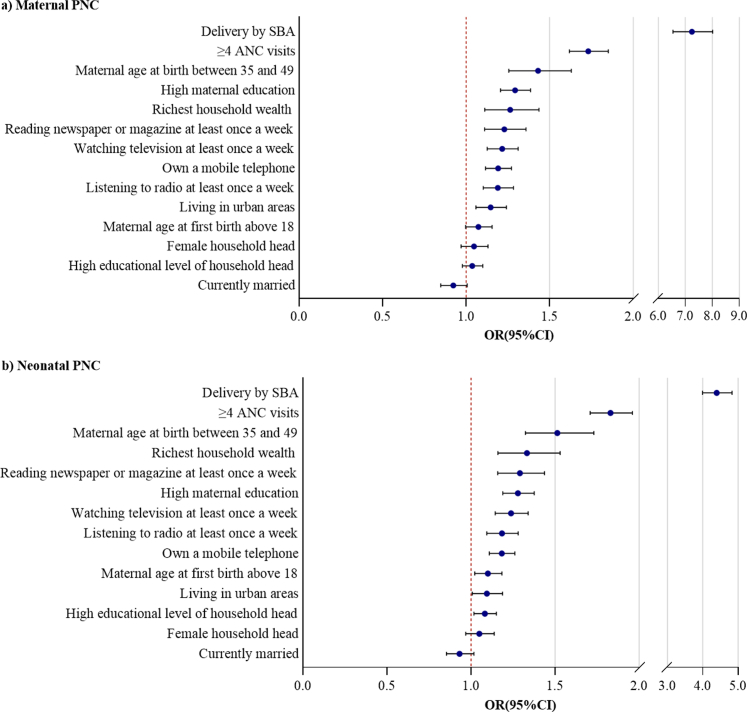
**Relative ranking of 14 factors associated with quality PNC from supplementary analysis of fully adjusted models on maternal care and neonatal care, excluding institutional delivery.** Note: High maternal education indicates the woman received secondary education or above; High education level of household head indicates the head of the woman’s household received secondary education or above; PNC indicates postnatal care; ANC indicates antenatal care; SBA indicates skilled birth attendant; OR indicates odds ratio.

**Appendix Fig. S11:**
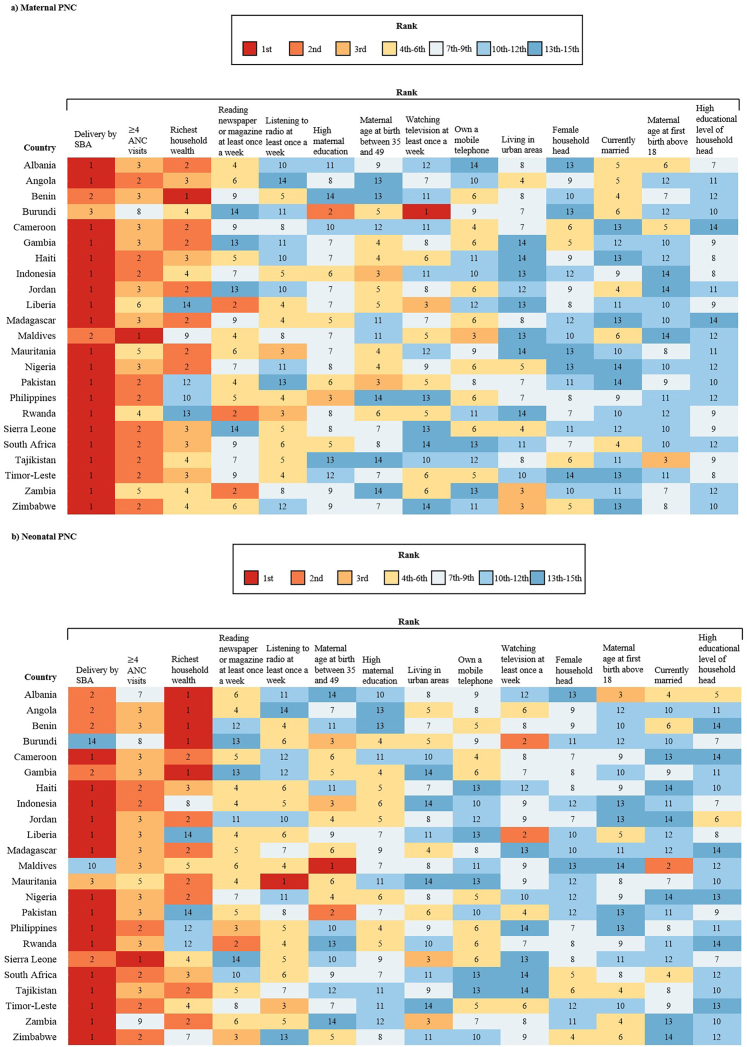
**Country-specific ranking of 14 factors associated with quality PNC from supplementary analysis of fully adjusted models on maternal care and neonatal care in 23 Countries, excluding institutional delivery.** Note: High maternal education indicates the woman received secondary education or above; High education level of household head indicates the head of the woman’s household received secondary education or above; PNC indicates postnatal care; ANC indicates antenatal care; SBA indicates skilled birth attendant; OR indicates odds ratio.

Finally, we excluded delivery by SBA and institutional delivery from the fully adjusted models to avoid potential multicollinearity. The rankings and magnitudes of all factors remained largely consistent (Appendix Figs. S8–S11). However, when delivery by SBA was excluded, institutional delivery became the top risk factor for neonatal PNC (Appendix Fig. S8B). Moreover, all VIFs were less than 5, indicating relatively low multicollinearity (Appendix Table S1).

## Discussion

This study investigated the association between 15 risk factors and the quality of PNC for mothers and neonates based on the most recent nationally representative household surveys in 23 countries. In pooled analysis, four protective factors were identified: delivery by SBAs, institutional delivery, four or more ANC visits, and advanced maternal age. Our findings were consistent in primary, subgroup, and supplementary analyses as well as across most countries. In country-specific analyses, the ranking of household wealth fell between third and fourth place, showing significant positive association with high-quality PNC for mothers and neonates.

We found that while institutional delivery was significantly associated with quality neonatal PNC in a single-adjusted model, it was less important when delivery by SBAs was included. While it is unsurprising that institutional delivery was found to play an important role in quality neonatal PNC, its diminishing effect may be explained by the fact that health facilities often do provide SBA services.^23^^,^^24^

Although institutional delivery was less important than delivery by SBAs for high-quality neonatal PNC, both institutional delivery and delivery by SBAs were found to be strongly associated with quality maternal PNC in our study. This difference may be due to the fact that institutional deliveries have been found to be linked with greater attention from SBAs for mothers, which in turn may increase the likelihood of receiving maternal PNC. Past studies have found that mothers tend to receive less attention than their neonates after delivery in at-home settings.^25^^,^^26^ This may be because neonates are often centred during the postnatal period and, as such, are more likely to receive postnatal services. Indeed, a study by Kim et al. indicated that neonates are more likely receive quality PNC when SBAs are present, regardless of where they are born.^27^ In contrast, mothers may require a specific institutional setting with SBAs present to receive quality PNC. This may explain why both institutional delivery and delivery by SBAs were found to have a significant impact on women's access to high-quality postnatal care in the regression models, whereas institutional delivery had a smaller impact than delivery by SBAs on quality neonatal PNC.

Our results also showed that wealthier households, more ANC visits, and advanced maternal age were positively associated with high-quality PNC for mothers and neonates. Compared with those from wealthier households, women from poorer households often have more limited health knowledge and less access to essential health services, including PNC.^23^^,^^28^^,^^29^ In addition, attendance of four or more ANC visits was found to be strongly associated with higher quality of maternal and neonatal PNC, as there is an increased opportunity for mothers to become more informed about potential complications and receive greater encouragement to seek high-quality PNC.^30^ Moreover, our study found that advanced maternal age was associated with quality PNC, which was consistent with the findings of studies conducted in Nepal and Uganda.^31^^,^^32^ Older women are often considered a high-risk group and perceived as requiring greater attention and quality PNC due to increased risk of maternal and neonatal mortality.^33^ While Perera et al. found advanced maternal age to be associated with lower PNC in Sri Lankan women, their study was only conducted in one country and may not be generalisable on a larger scale.

In addition, we found that exposure to mass media, possession of a mobile phone, and higher maternal education level were positively associated with high-quality PNC. Mass media has the potential to reach women with low levels of education and encourage them to use maternal health services.^34^ It may also have a similar effect on their partners, motivating them to encourage the women to seek care.^35^ Owning a mobile phone was also positively associated with quality PNC, as it can improve access to health information for women and facilitate timely and efficient communication with health providers.^36^^,^^37^ Similarly, as many studies have previously shown,^19^^,^^38^ women with higher education levels have higher odds ratios of seeking PNC services, since they are generally equipped with more health knowledge.^39^

There were several limitations in our study. Firstly, although our study used the widest and most recent available data, the results may not be generalised globally, as only 23 countries were included in the present study. Secondly, the fully adjusted models may have included factors that were associated with one another and which may have acted as confounders or mediators. However, our supplementary analysis showed low multicollinearity. Thirdly, the self-reported nature of DHS data may have introduced measurement and misreporting errors. To mitigate this, we applied rigorous analytical procedures during estimation and conducted supplementary analyses to address this issue. Lastly, a cross-sectional study design was used, which does not allow for causal inference. Further studies should explore the causal relationships between risk factors and quality PNC. Nonetheless, our study's use of the latest data from each of the included countries and inclusion of both maternal and neonatal PNC provided valuable insights into the provision of care for the mother-baby dyad. By examining the quality of PNC rather than merely PNC coverage, this study offers deeper insights and guidance for future research in this area.

Based on up-to-date data from 23 countries, our study revealed that women who received assistance from SBAs during delivery, delivered in institutional settings, were at an advanced maternal age, were from wealthier households, and attended four or more ANC visits were more likely to receive quality PNC for themselves and for their neonates. The relative importance of all factors was weaker and more heterogeneous across the analysed countries. These results highlight the importance of targeting the most significant factors associated with high-quality PNC through effective policies and programmes.

## Contributors

SY, YX, AX, QW and KT provided overall supervision of the study. SY, YX and QW conceptualised and designed the study. SY led the data analysis. SY and YX did the data interpretation and wrote the initial manuscript. AX, QW and KT were responsible for editing and proofreading the manuscript. SY, QW and KT had full access to all of the data in the study and take responsibility for the integrity of the data and the accuracy of the data analysis. SY, YX and QW accessed and verified the underlying data. All authors contributed to the critical revision of the manuscript for important intellectual content. All of the authors read and approved the final version of the manuscript. The corresponding authors had full access to all the data in the study and the final responsibility to submit the manuscript for publication.

## Data sharing statement

The data used in the analyses is publicly available upon request from the DHS. More information on DHS and access to the survey datasets can be found at https://dhsprogram.com/.

## Declaration of interests

We declare no competing interests.
